# Classroom aerosol dispersion: desk spacing and divider impacts

**DOI:** 10.1007/s13762-021-03564-z

**Published:** 2021-07-30

**Authors:** P. Dacunto, D. Moser, A. Ng, M. Benson

**Affiliations:** 1 Department of Geography and Environmental Engineering, United States Military Academy, 745 Brewerton Road, West Point, NY 10996 USA; 2 Department of Civil and Mechanical Engineering, United States Military Academy, West Point, NY 10996 USA

**Keywords:** Aerosol, Dispersion, COVID-19, Classroom air quality, Proximity effect, Desk dividers

## Abstract

A study of aerosol dispersion was conducted in a university classroom using a CO_2_ tracer gas emitted from three source locations in a steady release, one source location per test. The tracer gas emitted from the single source location represented the potentially infectious aerosol droplets emitted from a single student and was thus a way to examine the influence of one sick student on the rest of the class. Two parameters were adjusted during the testing—the spacing of the desks, which included a spread and compressed configuration, and the inclusion of three-sided clear dividers attached to the student desk surfaces. Tracer dispersion was measured through the use of monitors in 13 locations within the classroom, with eight monitors representing seated student locations, four monitors representing a standing instructor along the classroom front, and one monitor at the return vent in the ceiling. As expected, spacing strongly influenced concentration levels at desks adjacent to the source location. The use of dividers reduced overall student and instructor location tracer concentrations when compared to desks without dividers in most cases. Finally, the influence of air change differences on the results was noted with consistent trends. The experimental construct provides a systematic means for classroom testing that may be broadly applicable to various configurations of classrooms beyond the one tested.

**Graphic abstract:**

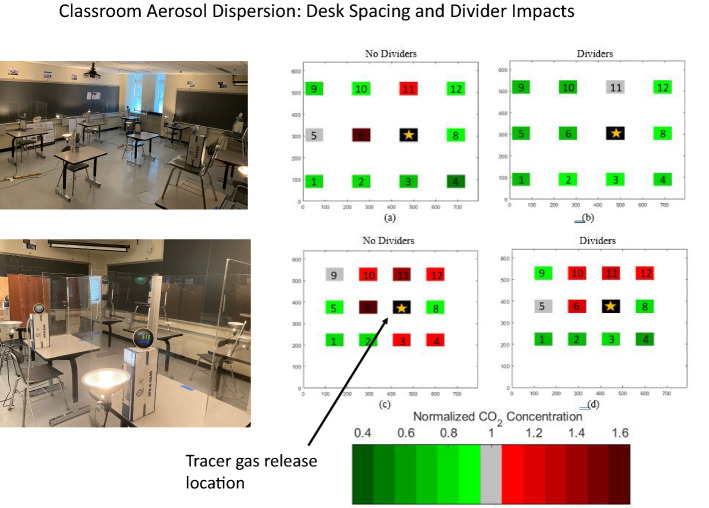

## Introduction

The rapid emergence of the novel coronavirus (SARs-CoV-2 or COVID-19) has drastically altered life in much of the world, halted the global economy, and forced schools and universities worldwide to transition to remote learning. Initial reports began surfacing in early January 2020 of a mystery illness spreading in China (Wee and Wang 2020); by March 11, the World Health Organization declared it a global pandemic (2020) with the initial outbreak in the US well underway (Gamio et al. 2020).

Research indicates that exhaled respiratory particles are a major transmission pathway (Morawska and Cao 2020; Jayaweera et al. 2020), both in the form of large droplets and much smaller aerosols expelled via coughing, sneezing, talking, and breathing (Papineni and Rosenthal 1997; Morawska 2006). Of particular concern for indoor air quality are these aerosols, which due to their small size (roughly less than 5 µm in diameter) are small enough to remain suspended in the air for hours (Morawska and Cao 2020; Morawska 2006). As a result, these aerosols are able to be inhaled and enter the lower respiratory tract, potentially causing infection (Robinson et al. 2012; Nicas et al. 2005).

The resulting exposures from both the aerosols and the larger droplets present a challenge to reopening in-person teaching in classrooms of schools and universities alike, where desks are often spaced closely and adequate ventilation may not exist. In addition, virus shedding tends to increase through speech (Asadi et al. 2019), creating an increased hazard as instructors lecture and students participate via questions or discussion. While non-pharmaceutical interventions (NPIs) such as the wearing of masks and physical distancing can help to reduce exposure to some of the larger droplets (Morawska and Cao 2020; Asadi et al. 2019), the aerosols remain a hazard.

Sufficient ventilation with fresh air is one of the most effective methods of limiting the transmission of viruses from suspended aerosols exhaled by an infected person (Qian and Zheng 2018). Indeed, the American Society of Heating, Refrigerating, and Air Conditioning Engineers (ASHRAE 2001) described changes to mechanical ventilation systems that can reduce airborne exposure to viruses in a guide for reopening schools after shutdown due to the pandemic (ASHRAE 2020). However, simply increasing the ventilation rate may not be possible for many buildings, and by itself may not be completely effective in reducing exposure. Other interventions must be considered to safely reopen classrooms.

This experimental study aimed to characterize the effects that desk spacing and transparent three-sided desk dividers had on reducing aerosol exposure from a single sick student sitting at multiple locations within a classroom. Increasing physical spacing aids in reducing exposure to the larger respiratory droplets (Morawska and Cao 2020; Asadi et al 2019). In addition, previous research has shown a “proximity effect” in which exposure to gases and particles at close range to an active source can be significantly higher than the concentration expected in the well-mixed portion of the room at large (Acevedo-Bolton et al. 2012; McBride et al. 1999). This is the result of non-instantaneous mixing, manifesting itself in short duration peaks known as “microplumes.” Acevedo-Bolton, et al (2012) found that 5-min average concentrations of a tracer gas within 1 m of a source varied more than a 100-fold, and that average concentrations within 0.25 m of the source were 6–20 times higher than the predicted well-mixed concentration.

In addition, three-sided desk dividers have obvious utility in blocking larger droplets from a cough or sneeze, but up to this point, their impact on aerosols has not been well characterized. Dividers placed on desks have been used in classrooms for various educational purposes, but limited research has been conducted on their influence on airflow within a classroom. Preliminary studies to characterize the effect of internal partitions on indoor airflow and contaminant distribution indicate that 1 m baffles lead to great changes in indoor airflow distribution (Liu et al. 2018). Additionally, Lee and Awbi (2004) analyzed internal partitions in a model test chamber to measure effects on indoor air quality and ventilation performance. Desk dividers may influence airflow within a classroom in a similar manner.

This study hypothesizes that increasing desk spacing will reduce exposure to aerosols emitted from a single source location within the room (i.e., breathing, coughing, sneezing, or talking from a sick student). Furthermore, it proposes that adding dividers will influence the airflow in such a way as to reduce aerosol exposure to fellow students and the instructor, in addition to stopping the larger non-aerosol droplets by acting as a physical barrier. In order to test these hypotheses, an inert tracer gas (carbon dioxide) was emitted from various source locations within a classroom (one source location per test, representing aerosols from a single sick student) as a proxy for the smaller infectious particles that will follow the streamlines. The resulting concentrations of tracer gas, as measured at various locations throughout the classroom with stabilized supply and return airflow conditions, enabled an evaluation of the impact of the various spacing and divider configurations. Experiments were conducted at a university in New York State, USA in summer 2020.

## Materials and methods

Testing occurred in a dedicated university classroom over three total days. A total of 12 different cases combining three different source locations, two spacing configurations, and two desk divider configurations were tested on day 1, and these same 12 cases were tested again on day 2 to check repeatability of results. On day 3, all three of the cases with desks spread and no dividers were tested again, each twice in a row, to further verify repeatability of results, and a collocation test of all instruments with a reference standard was conducted. In addition, tracer decay studies were conducted at the beginning and end of days 1 and 2, and at the beginning of the shorter day 3, to determine the air change rate.

### Classroom testing

**Fig. 1:**
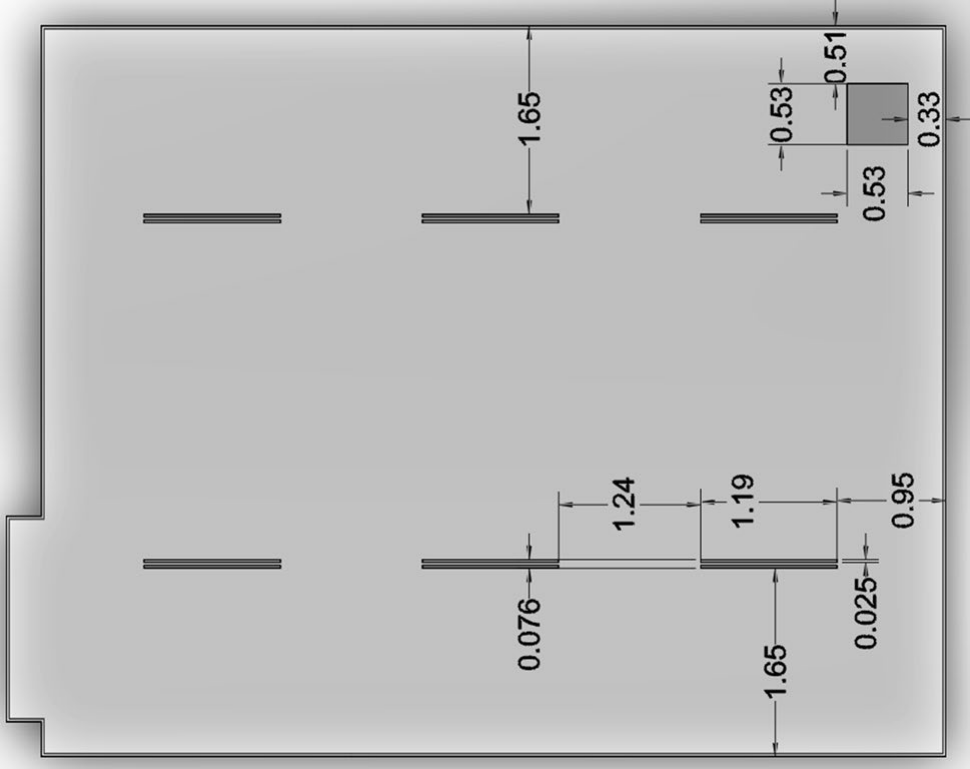
Ventilation supply and return dimensions and location in the classroom ceiling. Ventilation was provided from an air handling unit into the classroom via six rectangular supply vents and a square return vent in the front (upper right) corner, all in the ceiling. All dimensions in the figure are in meters

All tests were conducted in the same 7.9 m × 6.4 m classroom. The height of the room was 2.6 m, with an additional 0.9 m plenum space above the acoustic tiles housing the Variable Air Volume (VAV) box and ducting. Multiple anemometer measurements of the single small airflow path linking the plenum to the classroom indicated no net flow between the two zones. The ventilation system (Fig. 1) included six supply vents and one return vent, all in the ceiling. Because the single return vent was located in the front right corner, an asymmetric flow field was present in the classroom. The single door and two windows were closed to prevent additional airflow into the room, and the blinds were shut to minimize solar influence on the classroom temperature.

The six supply registers in the ceiling were 1.19 m long by a total of 0.08 m wide. Each register had a central divider placed longitudinally that was one third the overall width. This left two openings, each 0.025 m in width, each of which were further divided into two by a narrow vertical divider. Thus, the supply air left each register in four narrow vertical streams at a calculated velocity of approximately 0.8 m/s (based on the overall supply rate data from the VAV box, and the total area of the register openings).

The Air Handling Unit (AHU) for the classroom also serviced other classrooms and spaces throughout the building (a total of 67 VAV boxes). For the testing days, a technician sets the supply flow from the VAV box to a consistent setting, with automated data logs indicating the flow rate at 17.6 ± 0.4 and 17.7 ± 0.5 m^3^/min on day 1 and day 2 of testing, respectively. There was only one return fan on the AHU, which was automatically set at 5% below the AHU supply fan’s constant setting of 70%. It should be noted that individual classroom control of the return was not possible, and thus pressure variations in other classrooms could potentially impact the return in the testing classroom due to system balancing; however, the building was lightly occupied at the time of testing, and tracer decay tests (described below) indicated the impact within each day to be minimal. The pressure difference under the door at the beginning of each test was measured by a TSI DP-Calc 5815 micromanometer (TSI Inc, Shoreview, MN USA) and was relatively consistent, ranging from 1.5 to 2.2 Pa (day 1) and 1.2 to 2.1 Pa (day 2).

**Fig. 2:**
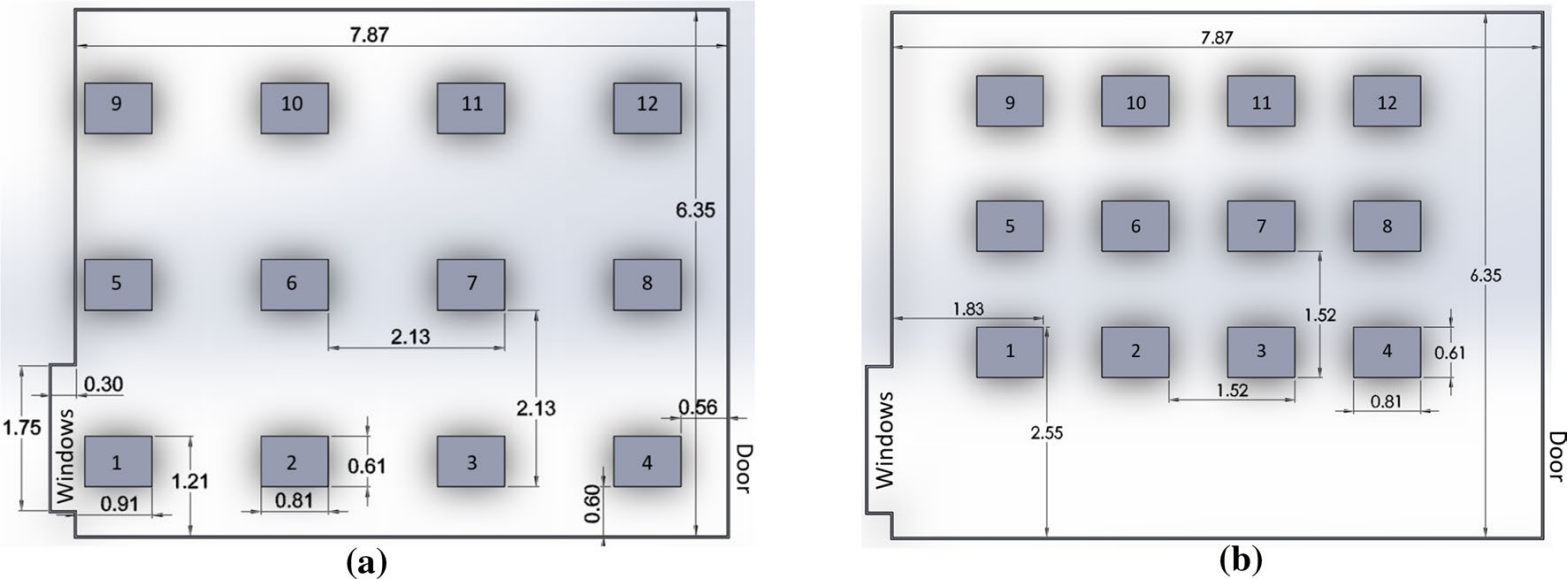
Classroom dimensions and desk locations. **a** Spread-out, **b** compressed. Positions 1–8 represented student locations, while positions 9–12 (at the front of the classroom) represented instructor locations. Two windows were at the cutout in the lower left, and a single door was at the lower right. All dimensions are in meters

**Fig. 3:**
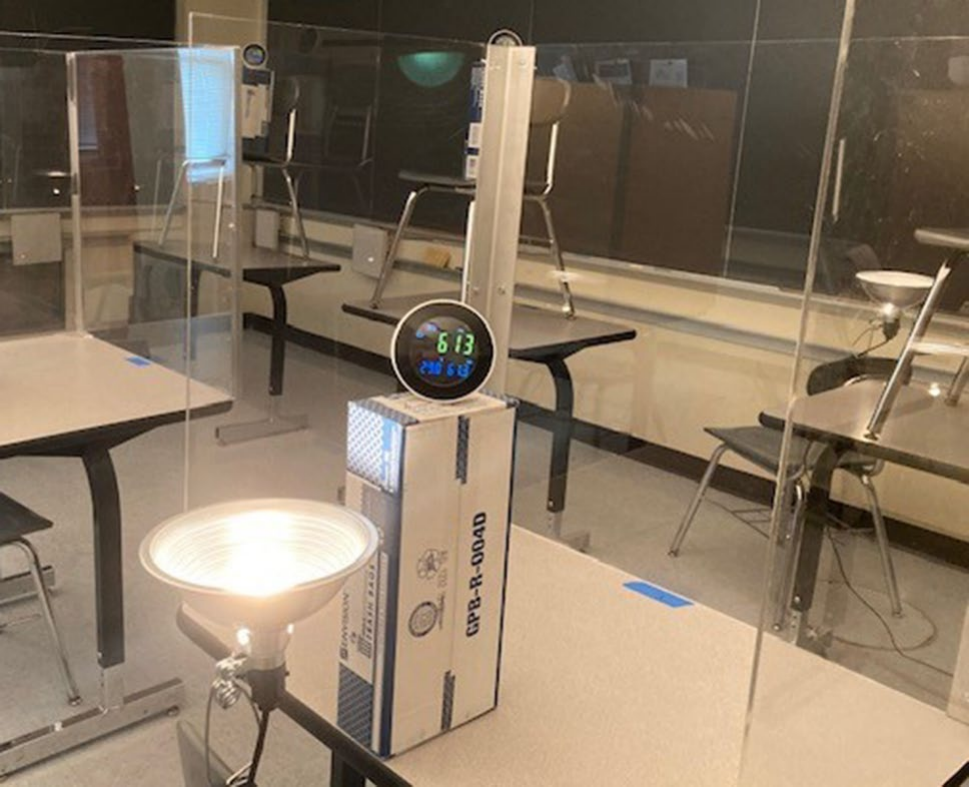
Desk dividers. A sample test setup. In the foreground is Desk 7 (pictured XT-10 CO_2_ monitor) with its associated heat lamp. Visible in the background are instructor stations 9–11. Only one lamp was used for the instructor and was placed in the middle of the instructor row

The desks were arranged in two configurations, a spread-out setup and a compressed setup as shown in Fig. 2. In the spread-out configuration, the desks were located 2.1 m apart (center to center), and in the compressed configuration, the desks were located 1.5 m apart. The arrangement consisted of eight “student” desks (#1–8), along with four additional “instructor” desks (#9–12) toward the front of the room. The instructor desks represented various locations where an instructor might be standing during class and were similar to the other desks except for the height of the monitor placed upon them. The compressed configuration maintained the same y-position for the instructor row (desks 9–12), along with the same center location. Additionally, a 100 W light bulb was placed approximately 10 cm behind the center of each student desk at a height of approximately 1 m to represent the thermal energy released by an average, seated university student (ASHRAE 2001). A 100 W light bulb was also placed at the center of the room, between positions 10 and 11, to represent the heat from the instructor. In six of the 12 test configurations, three-sided transparent acrylic “desk dividers” were placed on each of the student desks. These dividers extended to the edge of the 0.6 m by 0.8 m desks and were 0.7 m tall with the open side toward the location where the student would sit. The condition of desks compressed with no dividers represented the “baseline” case to which the impact of our two treatments (dividers and increased spacing) could be compared, since it was a more normal classroom setting. See Fig. 3.

### Carbon dioxide instrumentation

Thirteen carbon dioxide monitors were employed in each test: a single monitor on each of the 12 desks, and one hanging just below the center of the return to measure the CO_2_ concentration exiting the classroom, which was assumed to be reasonably representative of the well-mixed portion of the classroom. Monitors 1–8 were located at a height of 1.1 m to replicate the breathing zone of a seated student at each desk, and monitors 9–12 were at 1.5 m to replicate the breathing zone of a standing instructor. On day 1, all monitors were XT-10 indoor air quality monitors (CO2Meter.com, Ormond Beach, FL USA) except for monitor 12, which was an IQ-610 Indoor Air Quality Meter (Graywolf Sensing Solutions, Shelton CT USA). On day 2, Monitor 5 was replaced with an IQ-610 as well. All monitors were calibrated at the beginning of each day according to manufacturer’s specifications and were collocated for a period of 10–15 min to compare monitor responses. In addition, monitor clocks were synchronized, and logging intervals were set to 10 s.

On day 3, an additional collocation of all monitors was performed with an EGM-5 Portable CO_2_ gas analyzer (PP Systems, Amesbury MA USA), which had just been calibrated by the manufacturer. With an accuracy of < 1% over the calibrated range and an ability to auto-zero throughout the sampling period, the EGM-5 was able to serve as a reference instrument to which the other 13 monitors that had been used on day 1 and day 2 could be compared, with results adjusted accordingly as applicable. The collocation consisted of a 24 min period of sampling the typical indoor background concentrations (~ 500 ppm), followed by a CO_2_ tracer study of the classroom in which concentrations decayed from 1050 ppm to background. Linear regression of plots of the EGM response vs the XT-10 or IQ-610 responses during the tracer decay period revealed consistent linear relationships (*R*^2^ = 0.994, *n* = 1570 and *R*^2^ = 0.996, *n* = 314, respectively).

### Conduct of testing

Throughout each of the 30-min tests for the 12 different cases, CO_2_ tracer gas was released into the room at a constant rate from a cylinder in the hallway through 1.3 cm diameter soft plastic tubing under the door to the release location, where it widened to 2.5 cm diameter for the final 0.9 m so that the gas mixture was released at 1.2 m/s, a common exit velocity for both mouth and nasal breathing according to Tang et al. (2013). The release height was 1.1 m, at an angle that was approximately 15 degrees upward from the horizontal, to replicate the exhale location of an average student. The gas was a mixture of 75% N_2_ and 25% CO_2_ by volume to match the density of air, so that buoyancy was not a consideration. An in-line flowmeter (Omega FMA-1613A, Norwalk, CT USA) reported mass flow rates, which an operator adjusted via the regulator on the cylinder to keep the flow rate at a specified target of 0.94 g/s. This gave a steady-state concentration of approximately 800–900 ppm at the return, given the typical air exchange rates of the classroom (see below). This target steady-state concentration value was chosen to create a strong enough signal over background to create significant results, but not so much as to require logistically challenging quantities of tracer gas.

Tracer gas was released from a single location during each test (vs every location simultaneously) because of the study’s aim to examine the influence of aerosols released by a single sick student on the rest of the room; with only one tracer gas, releasing from all desks simultaneously would have made it impossible to determine the impact of the various release locations. Release locations were chosen at desks 1, 6, and 7 to be integrated into the different test cases. Desk 1 represented a “worst-case” scenario, since it was farthest from the return, while desks 6 and 7 were centrally located and could potentially have the influence on the greatest number of monitoring stations surrounding them. These three release locations combined with the two desk configurations (spread and compressed) and the two divider configurations (with and without) comprised the 12 test cases as listed in Table 1.

**Table 1:** Tests conducted on each day

Test #	Release source	Dividers test	Classroom configuration
1	Desk 1	No dividers	Compressed
2	Desk 1	No dividers	Spread
3	Desk 1	Dividers	Compressed
4	Desk 1	Dividers	Spread
5	Desk 6	No dividers	Compressed
6	Desk 6	No dividers	Spread
7	Desk 6	Dividers	Compressed
8	Desk 6	Dividers	Spread
9	Desk 7	No dividers	Compressed
10	Desk 7	No dividers	Spread
11	Desk 7	Dividers	Compressed
12	Desk 7	Dividers	Spread

Tracer decay tests to measure air change rate consisted of a short period of release of a large enough quantity of gas near the center of the room to achieve a well-mixed concentration of 1000–1200 ppm. During and just after the gas release, fans and other methods were used to mix the gas uniformly through the room. Dividers were not present during tracer decay tests, to further enhance mixing. Once relatively uniform concentrations were achieved at all monitoring locations, we turned off the fans, left the room, and let CO_2_ concentrations exponentially decay to background. In each study, linear regression of a plot of the natural logarithm of the background-subtracted concentrations of monitor 13 (at the return) vs time yielded a line (*R*^2^ > 0.99) whose slope was the air change rate (Laussman and Helm 2011). See Table 2.

**Table 2:** Air exchange and ventilation rates

Test #	Air change rate (h^−1^)	Ventilation rate (m^3^/min)
Day 1 AM	8.5	18.6
Day 1 PM	7.9	17.3
Day 2 AM	9.6	21.0
Day 2 PM	10.6	23.1

Ventilation rates were calculated using the measured air change rate and the classroom volume

### Data analysis

Data analysis was based on mean concentration responses of each monitor from minutes 20–30 of each test. This represented an approximate steady-state condition; indeed, a mass balance model of constant tracer release indicates that by the beginning of the period at minute 20, the concentration would theoretically be 93–97% of the way to steady state based on the air change rates measured in the room during the testing period.

To compare relative concentrations, each monitor’s equilibrium concentration value was normalized by the equilibrium value of monitor #13 at the return using Eq. (1)

1 $$CN_{i,n} = \frac{{c_{i,n} }}{{c_{13,n} }}$$

in which *CN*_*i,n*_ represents the normalized equilibrium concentration value for monitor *i* in test *n*, *c*_*i,n*_ is the raw CO_2_ equilibrium concentration value (ppm) for monitor *i* in test *n*, and *c*_*13,n*_ is the raw CO_2_ equilibrium concentration value (ppm) for monitor 13 in test *n.* This calculation gave one value for each location in each test that could be compared between tests, even if test conditions such as the air change rate varied slightly.

Raw monitor responses (*c*_*i,n*_) were adjusted based on the collocation conducted at the beginning of each day using an arithmetic offset. For use in Eq. (1), all monitor responses were not corrected to the EGM-5 with the relationship derived from the collocation on day 3, because slopes among the XT-10 and IQ-610 monitors were relatively similar and would essentially cancel out. However, monitor #13 results were adjusted to the EGM-5 reference concentration for the air change rate studies.

### Uncertainty

Sources of uncertainty in each test included the individual monitor response, the variability of the air change rate in the classroom, the rapidly changing nature of small-scale dispersive eddies and microplumes (Acevedo-Bolton et al. 2012) within the classroom volume during the equilibrium period, and the tracer gas supply flow rate (error of the monitor, as well as the ability to hold the flow steady at 0.94 g/s).

Collocation with the EGM-5 monitor on day 3 indicated that after applying the daily arithmetic offset, the XT-10 monitors were within 30 ppm of one another after 4 min into the decay period, when EGM-5 concentrations were changing at a rate less than 3% per 10 s. This eliminated an accounting of uncertainty due to slightly varying instrument response times, given that the concentrations measured during the test periods were relatively steady state, and that the data analysis was using average responses from that period. In addition, the IQ-610 monitors were also all typically within 20 ppm of each other and the XT-10 instruments during the same period; specifically, the average differences between responses of each IQ-610 and all the XT-10 s were 10 ± 8 ppm and 10 ± 1 ppm.

To minimize the variability of the air change rate in the classroom throughout each testing day, room ventilation conditions were held as constant as possible; as described in paragraph 2.1 above, supply flow rates were essentially the same on both of the primary testing days, with standard deviations within 3% of the mean and pressure differences under the door between the hallway and classroom within 0.9 Pa at the beginning of each test.

The primary measure of uncertainty was the standard deviation of the raw equilibrium concentrations (minutes 20–30) for each monitor in each test (σ_*ci,n*_), expressed as a relative error, S:

2 $$S_{{c_{i,n} }} = \frac{{\sigma_{{c_{i,n} }} }}{{c_{i,n} }}$$

This measure encompassed the variability of the small-scale air currents and microplumes within the classroom, as well as the tracer gas flow rate. In addition, it overshadowed the small uncertainty of the CO_2_ monitor responses; plots of the CO_2_ concentrations as measured by each monitor during the equilibrium timeframe indicate a periodic nature more reflective of the influence of small plumes and air currents than monitor noise.

Using standard methods for propagation of error (Meyer 1975), the relative uncertainty for the normalized monitor values in each test (*CN*_*i,n*_) could then be calculated using a root sum of squares of the relative uncertainties of monitor *i* and monitor 13:

3 $$S_{{CN_{i,n} }} = \sqrt {\left( {S_{{c_{i,n} }} } \right)^{2} + \left( {S_{{c_{13,n} }} } \right)^{2} } .$$

### Calculation of the C_Eval_ parameter

In order to assess the impact of our two treatments (addition of dividers and expansion of desk spacing) against our control (the baseline condition of desks in a compressed configuration with no dividers), the resulting *CN* values were analyzed in two ways. The first was to examine the sheer *quantity* of locations with *CN* more than 10% greater or less than 1.0 (i.e., < 0.9 or > 1.1), and the second was to examine the net *magnitude* of the increase or decrease in *CN* values throughout the room. In addition, spatial “heat” maps were created that gave an indication of which locations had higher or lower concentrations, and thus enabled a better understanding of the general flow characteristics of the room.

**Fig. 4:**
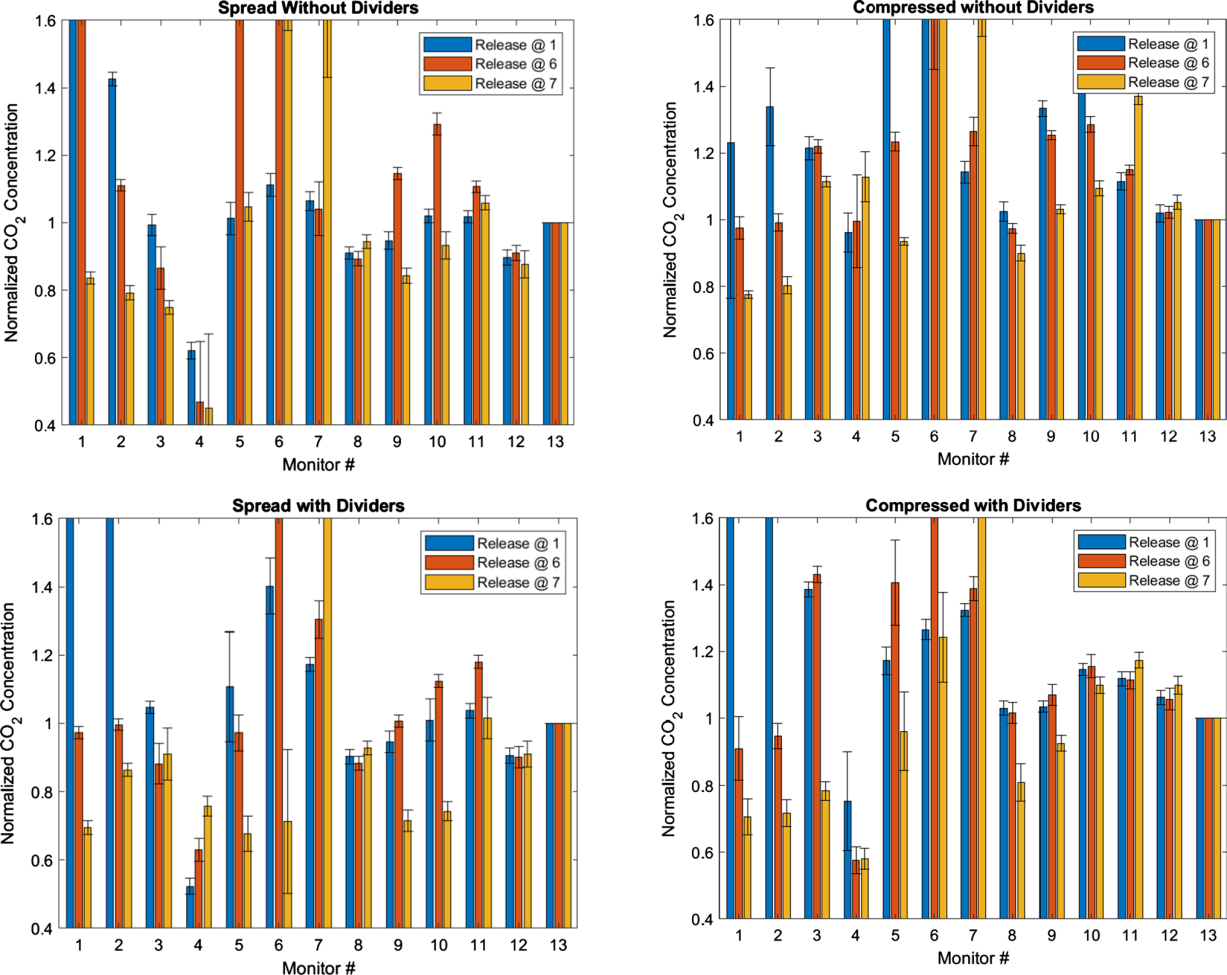
Normalized concentrations (*CN*) for all tests on day 2, in both spread and compressed configurations

The quantity of monitor locations with *CN* significantly greater than 1.1 or less than 0.9 could be visualized via bar graphs that display *CN* (with uncertainty bounds) for every monitor and release location in a given configuration (e.g., spread, no dividers); an example of these (day 2 results) is in Fig. 4, with a rollup of results from both days in Tables 3 and 4.

**Table 3:** Quantity of monitoring locations with *CN* significantly greater than 1.1 or less than 0.9

Configuration	Dividers	# Locations with *CN* < 0.9			# Locations with 0.9 < *CN* < 1.1			# Locations with *CN* > 1.1		
		Day 1	Day 2	Total	Day 1	Day 2	Total	Day 1	Day 2	Total
Compressed	No	1	1	2	18	17	35	14	15	29
Compressed	Yes	4	5	9	16	16	32	13	12	25
Spread	No	8	6	14	16	22	38	9	5	14
Spread	Yes	8	7	15	17	20	37	8	6	14

This table provides a summary of the 264 possible *CN* classifications over the two days of testing: four configurations per day, each with three release locations and 11 monitors throughout the room to categorize for each release location (monitor 13 and the monitor at the source location were not included). In order for a monitoring location to be counted as greater than 1.1 or less than 0.9, its uncertainty bounds had to exceed or be less than those parameters as well

**Table 4:** Quantity of monitoring locations with *CN* significantly greater than 1.1 or less than 0.9 for instructor locations only

Configuration	Dividers	# Instructor locations *CN* < 0.9			# Instructor locations 0.9 < *CN* < 1.1			# Instructor locations *CN* > 1.1		
		Day 1	Day 2	Total	Day 1	Day 2	Total	Day 1	Day 2	Total
Compressed	No	0	0	0	6	6	12	6	6	12
Compressed	Yes	0	0	0	7	9	16	5	3	8
Spread	No	3	1	4	4	9	13	5	2	7
Spread	Yes	2	2	4	7	8	15	3	2	5

This table includes a count of CN classifications for the instructor locations (monitoring locations 9–12) only

To account for the magnitude of the overall change in *CN* values throughout the room, a new parameter (*C*_Eval_) was created. This new parameter (*C*_Eval_) enables a holistic classroom comparison and compares a given condition to the “baseline” or control condition (the compressed, no divider configuration). Thus, *C*_Eval_ for test case *n* is defined as the following,

4 $$C_{{{\text{Eval}},n}} = \frac{{\left[ { \mathop \sum \nolimits_{i = 1}^{12} \left( {CN_{i,n} } \right)} \right] - \left( {CN_{{{\text{source}},n}} } \right)}}{{\left[ { \mathop \sum \nolimits_{i = 1}^{12} \left( {CN_{{i,{\text{baseline}}}} } \right)} \right] - \left( {CN_{{\text{source,baseline}}} } \right)}}$$

where *CN*_*i,n*_ is the normalized concentration of monitor *i* for test case *n*, *CN*_*i,*baseline_ is the normalized concentration of monitor *i* in the baseline case (same release location, desks compressed with no dividers), *CN*_source*, n*_ is the normalized concentration of the monitor at the source location, and *CN*_source, baseline_ is the normalized concentration of the monitor at the source location in the baseline case. The reason for removing the source test and baseline terms is that due to the proximity of the monitor to the release point, the terms were unrealistically high and added no information about the dispersion within the room. Monitor 13 was not included in the summation because it was the normalization parameter, and thus the term would end up as 1.0 in both the test and baseline case. A *C*_Eval_ greater than 1.0 indicates a higher (worse) overall concentration at the measurement locations in the room in the test case as compared to the baseline case, whereas a value less than 1.0 indicates a reduced (better) overall concentration condition.

Uncertainty in *C*_Eval_ was calculated through propagation of the relative error of the normalized concentration for each monitor as described above (*S*_*CN i,n*_). First, the absolute errors of the top and bottom terms of Eq. (4) were calculated as the root sum of squares of each *S*_*CN*_ value in the summation. Then, the relative error of *C*_Eval_ could be computed as the root sum of squares of the relative error of the top and bottom terms. The *C*_Eval_ with relative uncertainty (S_Ceval_) is presented for every experiment on both days in Tables 5 and 6.

**Table 5:**
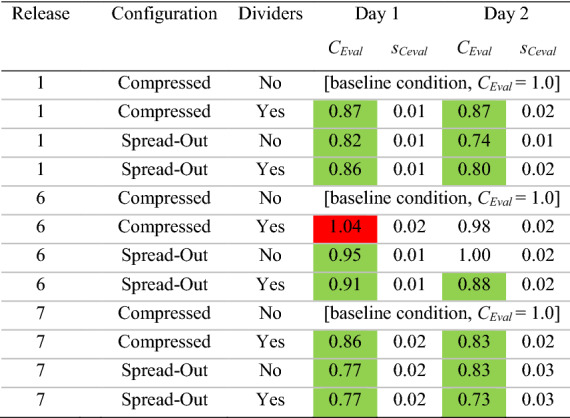
*C*_Eval_ results

The *C*_Eval_ ± *s*_Ceval_ parameter provides an assessment of the overall magnitude of departure of concentrations from the baseline case (same release location, compressed configuration with no dividers) at all monitoring locations throughout the room. A *C*_Eval_ greater than 1.0 represents a net increase in concentration at the monitoring locations (a worse condition) as compared to baseline, whereas a *C*_Eval_ less than 1.0 indicates a net decrease. *C*_Eval_ terms significantly greater than 1.0 (i.e., beyond the uncertainty bounds) are colored red; those significantly less than 1.0 are in green

**Table 6:**
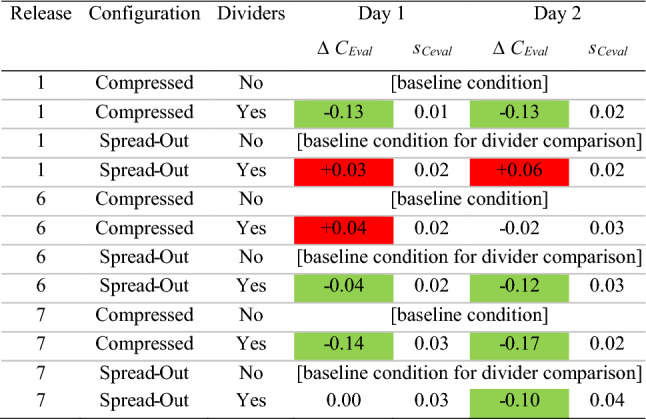
Change in *C*_Eval_ resulting from the addition of dividers

This table displays the change in *C*_Eval_ (Δ*C*_Eval_) when isolating for the impact of dividers. Thus, in each case, we compute the difference in the *C*_Eval_ parameter between the “without” and the “with” divider condition for a particular release location and desk configuration. Δ*C*_Eval_ terms indicating a significant increase in *C*_Eval_ from the “without” to the “with” divider condition are colored red; those with a significant decrease are in green. The uncertainty (*s*_Ceval_) of Δ*C*_Eval_ was calculated as the root sum of squares of the uncertainty of the two *C*_Eval_ terms

## Results and discussion

### Air change rate testing results

Table 2 shows the air change rates as determined by tracer decay and the corresponding ventilation rate based on the 131 m^3^ volume of the classroom. While the measured ventilation rates were very close to the flow rate of supply air in the VAV box on day 1 (17.6 ± 0.4 m^3^/min), on day 2, they were significantly higher even though the VAV box record showed an almost identical supply flow rate (17.7 ± 0.5 m^3^/min). This is attributed to variability in the return flow, which in turn was caused by varying conditions in other spaces served by the AHU and its requirement to balance flows. The fact that the ventilation rate was approximately 20% higher on day 2 than day 1 ended up being beneficial, in that replicability could be checked under slightly varying ventilation conditions.

### Impact of desk spacing

If one of the most effective non-pharmaceutical interventions for preventing the spread of COVID-19 is to physically distance, we would expect to see decreasing exposure to infectious aerosols (as represented by our CO_2_ tracer gas) with increasing distance between the receptor and the source location. This is demonstrated in both Fig. 4 (the example bar charts) and Tables 3 and 4; the quantity of monitoring locations with *CN* > 1.1 roughly halves from the compressed to the spread configurations, and the number of monitors with *CN* < 0.9 increases significantly. The effect is consistent across both days of tests, as well as both divider conditions.

In addition, the values of *C*_Eval_ (which take into account the magnitude of monitoring locations’ departure from the normalization concentration) also indicate that the locations throughout the room become holistically better as we expand the desk spacing. Table 5 indicates that 11 of 12 spread configuration values over the two days are significantly less than 1.0 (better than the compressed baseline case), with the other essentially unchanged. This is an intuitive result; spacing is well known to be a primary consideration in reducing exposure to aerosols (Acevedo-Bolton et al. 2012), and the data strongly support that conclusion.

**Fig. 5:**
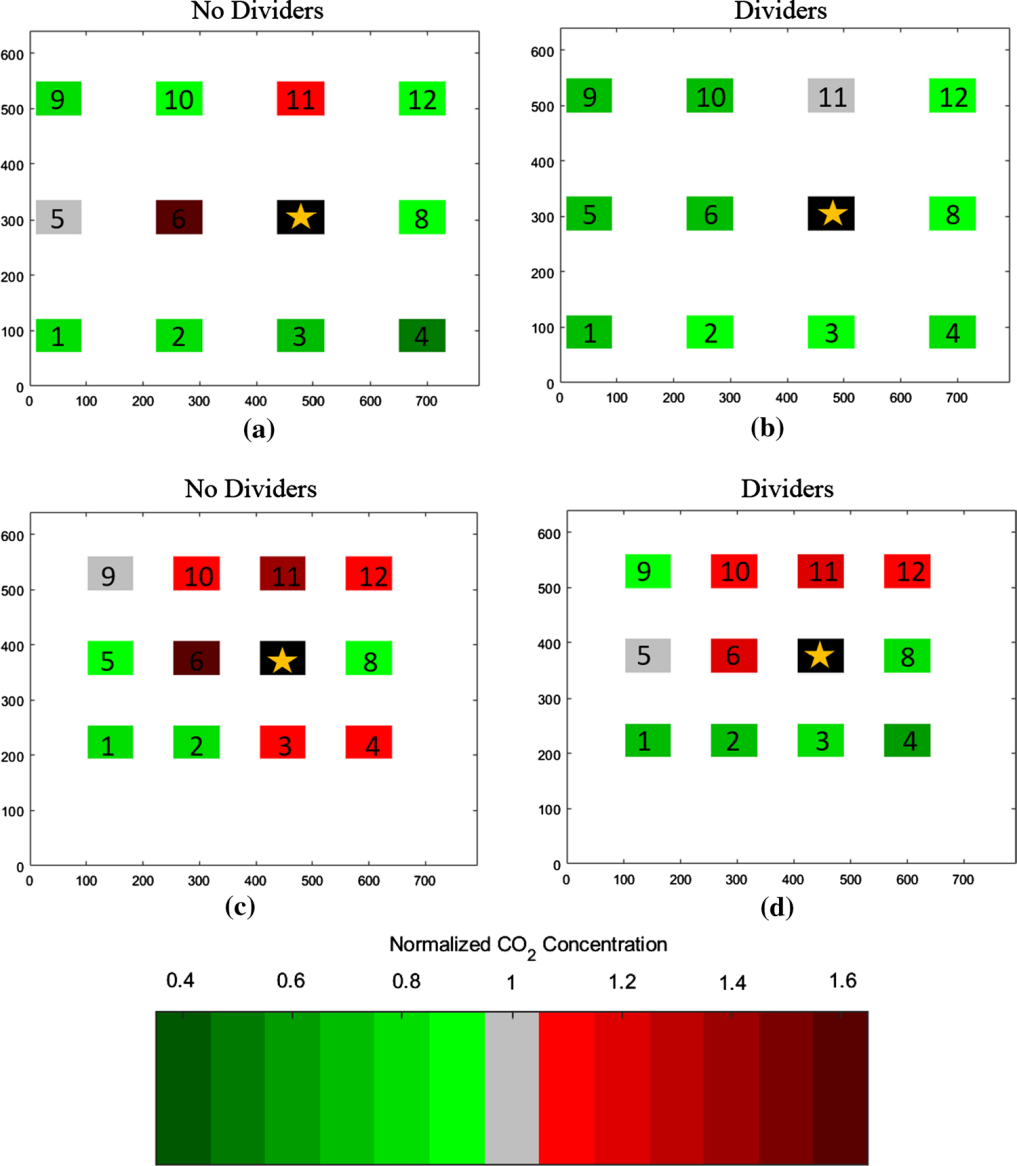
Spatial plots of normalized concentrations (*CN*) for desk 7 release (day 2): the release location is indicated in black. **a** Desk 7 release, spread, without dividers. **b** Desk 7 release, spread, with dividers. **c** Desk 7 release, compressed, without dividers. **d** Desk 7 release, compressed, with dividers. *X* and *Y* axes are in centimeters and help denote absolute locations within the room

**Fig. 6:**
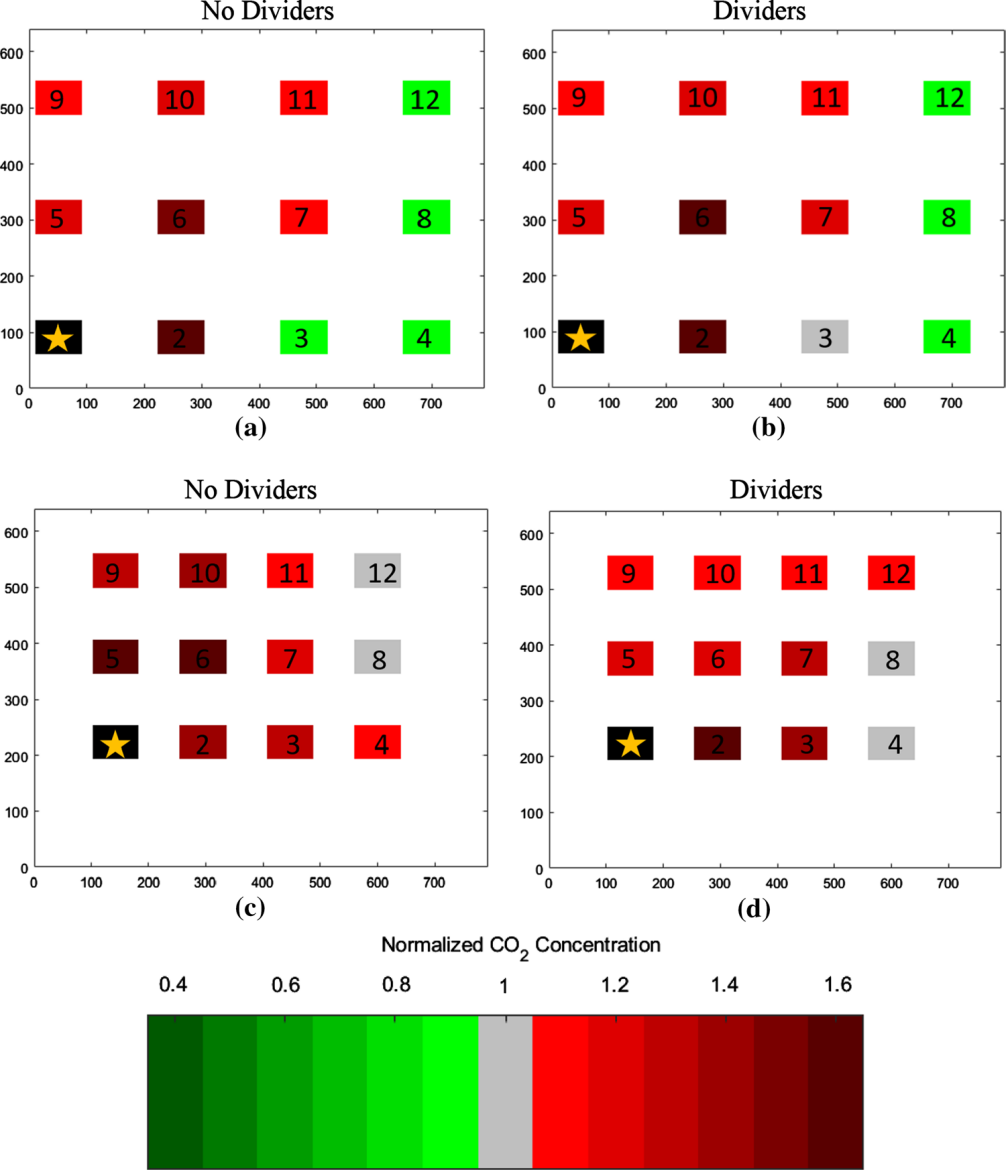
Spatial plots of normalized concentrations (*CN*) for Desk 1 release (day 1): the release location is indicated in black. **a** Desk 1 release, spread, without dividers. **b** Desk 1 release, spread, with dividers. **c** Desk 1 release, compressed, without dividers. **d** Desk 1 release, compressed, with dividers. *X* and *Y* axes are in centimeters and help denote absolute locations within the room

Finally, the spatial plots in Figs. 5 and 6 also show this effect graphically. As one compares the lower figures to the upper for both the desk 1 and desk 7 releases, it becomes quickly evident that the number of desks that are red (CN > 1.0) decreases dramatically in the spread configuration. In addition, these plots also show the general airflow from lower left to upper right toward the return.

### Impact of dividers

To isolate the impact of dividers, a comparison can be conducted of the results from with and without divider conditions in the spread configuration, and again in the compressed configuration. When analyzing by quantity of monitoring locations, Table 3 clearly indicates a consistent trend across both days of testing: when dividers are added, the impact is greater in the compressed than the spread configuration. In the compressed configuration, the addition of dividers causes the number of monitoring locations with *CN* < 0.9 to increase by 7, and the number with CN > 1.1 to decrease by 4. This is compared to the spread configuration, in which the number of monitors in either category is essentially unchanged.

Regardless, the above analysis does not take into account the magnitude of the improvement or reduction at each location in the room, and so the *C*_Eval_ parameter is a much better measure of the overall impact of dividers. As shown in Table 6, in 7 of the 12 cases (three release points, two desk spacing configurations over 2 days), the addition of dividers caused a significant reduction (improvement) in *C*_Eval_.

In 3 of the 12 cases, *C*_Eval_ slightly increased with the addition of dividers, and in two cases, it was essentially unchanged. Specifically, on both day 1 and day 2 of testing, *C*_Eval_ was significantly higher (worse) with a release location of desk 1 in the spread configuration. This phenomenon may have resulted from this being the only case where the release location was behind every ceiling supply vent; as a result, the tracer gas became relatively contained by the dividers in the back corner. This created a very high concentration on desk 1 (*CN* = 3.9 and 5.4 on day 1 and 2) and desk 2 (*CN* = 2.0 and 1.9) that also influenced the immediately adjacent desks 6 and 7 as well, contributing to an overall worse condition in the room. This impact could potentially be remedied by relocating those desks to a location in front of the supply vents, or other ventilation and configuration changes. Desk 6 compressed also had a minor but significant increase in *C*_Eval_ on day 1, but the parameter was essentially unchanged on day 2.

The spatial maps (Figs. 5, 6) shed additional light on these trends. Figure 5, which shows the desk 7 release on day 2, clearly shows a significant reduction in *CN* in the desks surrounding desk 7 in both the spread and compressed configurations. The dividers in the compressed configuration seem to prevent any dispersion to the back row, possibly because they serve to channel the tracer gas released at desk 7 to a high enough elevation in the room so that the emissions are caught in the general flow toward the return in the front of the room.

The desk 1 release in Fig. 6 shows a more muddled picture, given that the back row actually gets worse with the addition of dividers in the spread configuration; this is the only configuration where the addition of dividers increased *C*_Eval_ on both days of testing and as stated above may be because the dividers help to trap the tracer gas in an eddy behind the back row of supply vents. The compressed configuration shows a net improvement with the addition of dividers (since the back row has now moved under the supply vents), though all desks have a *CN* value equal to or greater than 1 in this worst-case scenario.

### Impact on instructor

Recognizing the air flow pattern of net forward advection from the supply to return vents in the classroom, an instructor at the front of the classroom is possibly more vulnerable to increased exposure than the bulk of the students present in the room. As such, the monitor impacts at the elevated positions 9–12 are of particular interest in the various configurations studied. Table 4 indicates that the total number of instructor locations with CN < 0.9 increases slightly when the desks are spread, and the number with CN > 1.1 decreases in both the spread and compressed configurations when dividers are added.

However, this analysis does not reflect whether or not there is an improvement or reduction in magnitude with dividers; for example, *CN* may remain greater than 1.0, but still be reduced. In addition, it does not provide a holistic assessment of the net improvement or reduction in the entirety of the instructor locations. An analysis of *C*_Eval_ for the instructor positions only in test *n* enables this analysis, by modifying Eq. (4) as such:

5 $$C_{Eval,n} = \frac{{\left[ { \mathop \sum \nolimits_{i = 9}^{12} \left( {CN_{i,n} } \right)} \right]}}{{\left[ { \mathop \sum \nolimits_{i = 9}^{12} \left( {CN_{i,baseline} } \right)} \right]}}$$

Note that, the subtraction of the source terms is no longer required, since the tracer release location was never at stations 9–12.

Tables 7 and 8 contain these results. By comparing Tables 7, 8 to Tables 5, 6 (*C*_Eval_ for the whole room), it becomes evident that the instructor positions fare slightly better than the room as a whole. Expanding desk spacing shows a similar pattern of reducing *C*_Eval_ to less than 1.0 in 11 of 12 cases, with it remaining essentially equal to 1.0 in the final case. In addition, the impact of dividers seems to be much more beneficial for the instructor stations, with 9 *C*_Eval_ decreasing significantly and none increasing out of the 12 possible comparison conditions over two days; this is compared to the *C*_Eval_ for the room as a whole, in which 7 *C*_Eval_ decreased and 3 increased. It seems that adding dividers channels the tracer gas up into the flow toward the return, essentially passing over the instructor’s “head” despite the higher monitoring location.

**Table 7:**
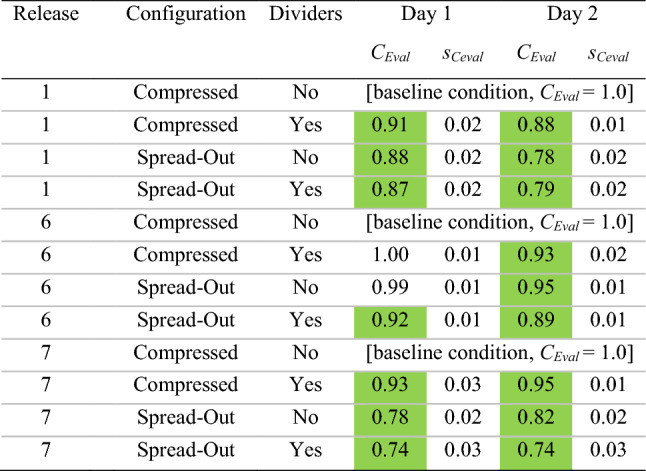
*C*_Eval_ for instructor desks (9–12)

In this table, *C*_Eval_ ± *s*_Ceval_ was computed for the instructor locations only. A *C*_Eval_ greater than 1.0 represents a net increase in concentration at monitoring locations 9–12 (a worse condition for the instructor) as compared to baseline, whereas a *C*_Eval_ less than 1.0 indicates a net decrease. *C*_Eval_ terms with a significant increase are colored red; those with a significant decrease are in green

**Table 8:**
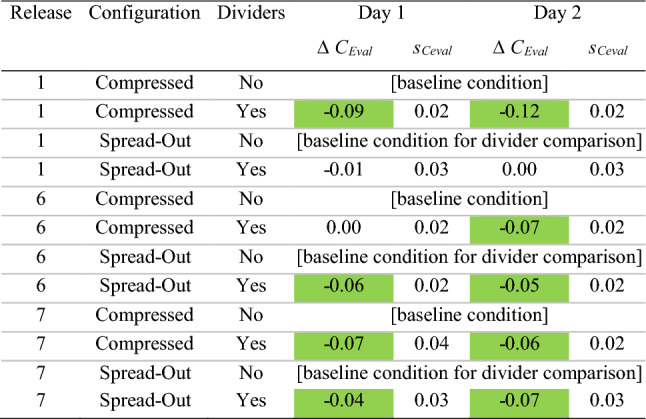
Change in *C*_Eval_ for instructor monitors (#9–12) resulting from the addition of dividers

This table displays the change in *C*_Eval_ (Δ*C*_Eval_) when isolating for the impact of dividers, for instructor locations only. Thus, in each case, we compute the difference in the *C*_Eval_ parameter between the “without” and the “with” divider condition for a particular release location and desk configuration. Δ*C*_Eval_ terms indicating a significant increase in *C*_Eval_ from the “without” to the “with” divider condition are colored red; those with a significant decrease are in green. The uncertainty (*s*_Ceval_) of Δ*C*_Eval_ was calculated as the root sum of squares of the uncertainty of the two *C*_Eval_ terms

Note that, for every release point for every spread configuration, monitor #12 (directly under the return) had a *CN* value that was consistently less than 1.0, but in every compressed configuration test, it was greater than or equal to 1.0 (see Fig. 4). This interesting result may be because the compressed geometry of the room changed it from a best case (below the net flow into the return) to a worst case (right in the path of the flow).

Of course, these instructor location results are based on a single orientation of the room, with the instructor teaching on the side of the ventilation air return (vicinity desks 9–12 as denoted in Fig. 2). Results would change significantly if the teaching axis of the room was changed so that the instructor was located along any of the other three walls. Based on the data in this study, it is difficult to say if there is a consistent trend for the other walls since one of the source locations (#1) would be located in an instructor location if the room was turned in two of the other directions. Also, in the compressed location, the back row of desks became pushed quite far away from the back wall (where an instructor would stand) as all desks were compressed toward the front of the room. More research is needed in this area.

### Will using dividers enable desks to be spaced more closely?

The *C*_Eval_ parameters in Tables 5 and 6 indicate that over the two days of testing, the addition of dividers in the compressed configuration resulted in a reduction in *C*_Eval_ in 4 of 6 cases, with it slightly increased in one and unchanged in the other. In addition, the “compressed with divider” configurations were worse than the “spread no divider” configurations in 4 of 6 cases and essentially unchanged in the other two. However, such comparisons between spread and compressed configurations are fraught due to the changes in absolute *x–y* desk locations within the room. While the relative positioning of monitors remained the same between the spread and compressed configurations, their absolute locations with respect to the HVAC supply and return vents had to change by necessity, and this certainly impacted the results.

Therefore, it is difficult to make a generalized comparison of relative concentration conditions between the configurations when both the spacing and divider conditions change simultaneously. While it cannot be concluded that dividers will enable desks to be spaced more closely with no increases in exposure, by isolating the impact of dividers (as in “Impact of dividers” section above), it can be said that if desk spacing does remain compressed, dividers will help.

### Impact of the air change rate on results

As discussed above, the air change rate increased by about 20% from day 1 to day 2. Despite this, the quantities of monitors with *CN* greater than 1.1 or less than 0.9 were relatively consistent from day 1 to day 2 (Table 3), and trends in *C*_Eval_ remained consistent between day 1 and day 2 (Table 5). This indicates that a modest but significant change in the air change rate did not have great impact on the overall results. However, the authors acknowledge that a more significant adjustment to the ventilation rate might have a larger impact.

### Limitations

The results described above are based on a single orientation of one classroom with a reasonably consistent air change rate. The design of the ventilation supply and return would impact air flow greatly, and thus further testing in a variety of different classrooms is required before any consistent trends can be noted for any classroom. For example, classrooms with fewer supply registers may have more problematic “dead zones” that result in areas of high concentration, and just rotating the teaching by 90 degrees could have a huge impact on certain locations. In addition, since the supply air had relatively low concentrations of CO_2_, these results may be more applicable to a classroom supplied by a dedicated single-zone ventilation system with filtration, versus one with poor filtration and recirculation into the supply air. The latter would result in higher concentrations overall, as the supply air would help to recirculate aerosols back into the room. Finally, the tracer gas represented the smaller aerosols that stay suspended for hours reasonably well, but not the larger particles which would be more influenced by settling. Nevertheless, this study provides some initial results that could inform future studies, as well as a testing protocol for future researchers to follow.

## Conclusion

This study utilized a tracer gas to assess the impact of desk spacing and dividers on aerosol exposure from a single sick student in a classroom. The *CN* and *C*_Eval_ parameters clearly show that spacing is of paramount importance; as desks were spaced farther apart, potential exposure decreased. The addition of dividers generally improved overall conditions in the room and was particularly beneficial for the instructor locations for this test scenario. In essence, the divider at the source location seems to function as a small chimney that routes the tracer gas (aerosol) upward toward the general flow of air through the room toward the return, assuming that the return is in the ceiling. They also seem to help to shield other locations from the aerosol, though the opposite effect of trapping the emissions could also occur if the desks are out of the general flowpath.

It should be noted that the measured concentrations do not have a direct correlation to viral load and do not tell us whether the exposure in a particular location is potentially infectious. However, they do give a sense of relative exposure, as well as the impact of the spacing and divider interventions.

Given the wide range of possible ventilation and classroom configurations, clearly, additional studies in other locations would be helpful to generalize these results. In addition, a study to determine the impact of the return location on the instructor’s exposure in particular would be very helpful. Regardless, this study indicates some clear trends regarding the impact of these two variables and provides a methodology for such work that can be potentially useful in further study.

## Data Availability

Available upon request of the authors.

## Code availability

Available upon request of the authors.
